# Reinstrumentation for rapid curve progression after implant removal following posterior instrumented fusion in adolescent idiopathic scoliosis: a case report

**DOI:** 10.1186/1748-7161-8-15

**Published:** 2013-09-03

**Authors:** Toshiaki Kotani, Tsutomu Akazawa, Jose MT Lumawig, Tsuyoshi Sakuma, Shohei Minami

**Affiliations:** 1 Department of Orthopedic Surgery, Seirei Sakura Citizen Hospital, 2-36-2 Ebaradai Sakura-shi, Chiba 285-8765, Japan; 2 Department of Orthopedic Surgery, The Medical City, Ortigas Avenue, Pasig City, Metro Manila 1605, Philippines

**Keywords:** Adolescent idiopathic scoliosis, Implant removal, Posterior spinal fusion

## Abstract

**Background context:**

Spinal implants are occasionally removed due to infection or soft tissue irritation secondary to prominence. Several studies have reported loss of scoliotic curve correction after implant removal. However, further review of the literature reveals no similar cases of rapid curve progression following implant removal in patients with adolescent idiopathic scoliosis (AIS) necessitating repeat posterior instrumented fusion.

**Purpose:**

To describe a 15-year-old female AIS patient treated by posterior instrumented fusion, who developed unusual rapid coronal and sagittal curve progression after implant removal.

**Study design:**

Case report.

**Methods:**

Retrospective case report.

**Results:**

A 15-year-old female with Lenke type 1A AIS underwent a successful posterior spinal fusion with instrumentation. She initially had no complications after surgery, but three years after instrumentation, her implants were removed due to pain secondary to implant prominence. Fifteen months after removal, the main thoracic curve increased, compared with radiographs taken before removal, from 29° to 57°. This development required the patient to undergo additional surgery, which involved multiple osteotomies and posterior reinstrumentation.

**Conclusions:**

We must acknowledge that a remarkable amount of progression can occur rapidly following implant removal in scoliotic patients. Taking this into consideration,we need to carefully explain to patients that removal of their implants entails a risk of progressive deformity and that they need to follow-up with their physician after implant removal.

## Background

Spinal implants are occasionally removed due to irritation secondary to prominent implants or infection in surgically-corrected scoliotic patients [1-3]. Despite the frequency of implant removal, relatively few reports in the literature have described the clinical and radiographic results following removal of posterior spine implants from scoliotic patients. Furthermore, to our knowledge, no cases have been reported of rapid progression of a coronal curve after implant removal (compared with the curve just prior to implant removal) to an extent requiring reinstrumentation in adolescent idiopathic scoliosis (AIS) patients.

We present the case of a 15-year-old young woman with AIS treated by posterior spinal fusion and instrumentation. Three years after instrumentation, implants were removed due to pain secondary to implant prominence. Fifteen months after removal, the main thoracic curve had increased significantly from 29° to 57°. This reemergence of scoliosis required multiple osteotomies and posterior instrumentation using pedicle screws inserted with navigation. The patient and her parents have given informed consent to submission of this case for publication.

## Case presentation

### History and physical examination

**Figure 1:**
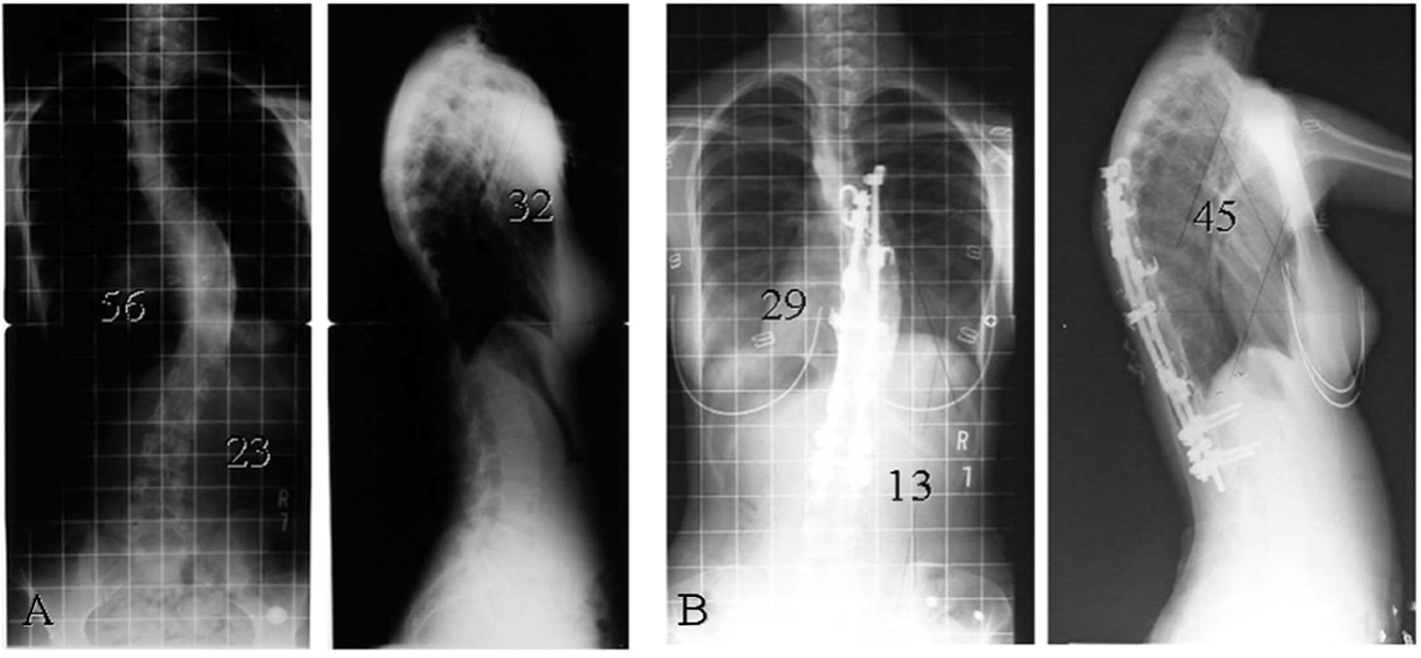
**Standing radiographs. (A)** Radiographs before initial surgery. **(B)** Radiographs 3 years after initial surgery.

A 15-year-old female diagnosed with AIS presented with a progressive curve classified as Lenke type 1A. The right thoracic Cobb angle was 56° (T6-T12), with a nonstructural left lumbar curve of 23° before her initial surgery (Figure 1A). She experienced menarche at age 12. She had an unremarkable past medical and family history and on physical examination, the forward bending test revealed a right thoracic hump. Her neurologic examination was normal. Magnetic resonance imaging and computed tomography (CT) myelogram showed no abnormal findings in her spine.

The patient underwent posterior spinal fusion (T6-L2) and three-level thoracoplasty (9-11th ribs) with 6.3 mm titanium rod and screw and hook instrumentation, sublaminar wiring, and autogenous rib and local bone grafting. The surgery substantially corrected her deformity, leaving her with a right thoracic scoliosis of 24°. She did well after the surgery until she started experiencing pain secondary to prominent implants in the upper thoracic spine one year after surgery. Three years after the initial surgery, at age 18, her back pain had worsened due to metallic prominence, and she wanted to remove her implants. We agreed to remove her implants, because our group had concluded at that time that the spinal implants should be removed after bony fusion to avoid corrosion of the spinal implants [4]. At the time of examination just prior to the planned implant removal, her Cobb angle was noted to have increased 5, to 29° (T6-T12) (Figure 1B).

**Figure 2:**
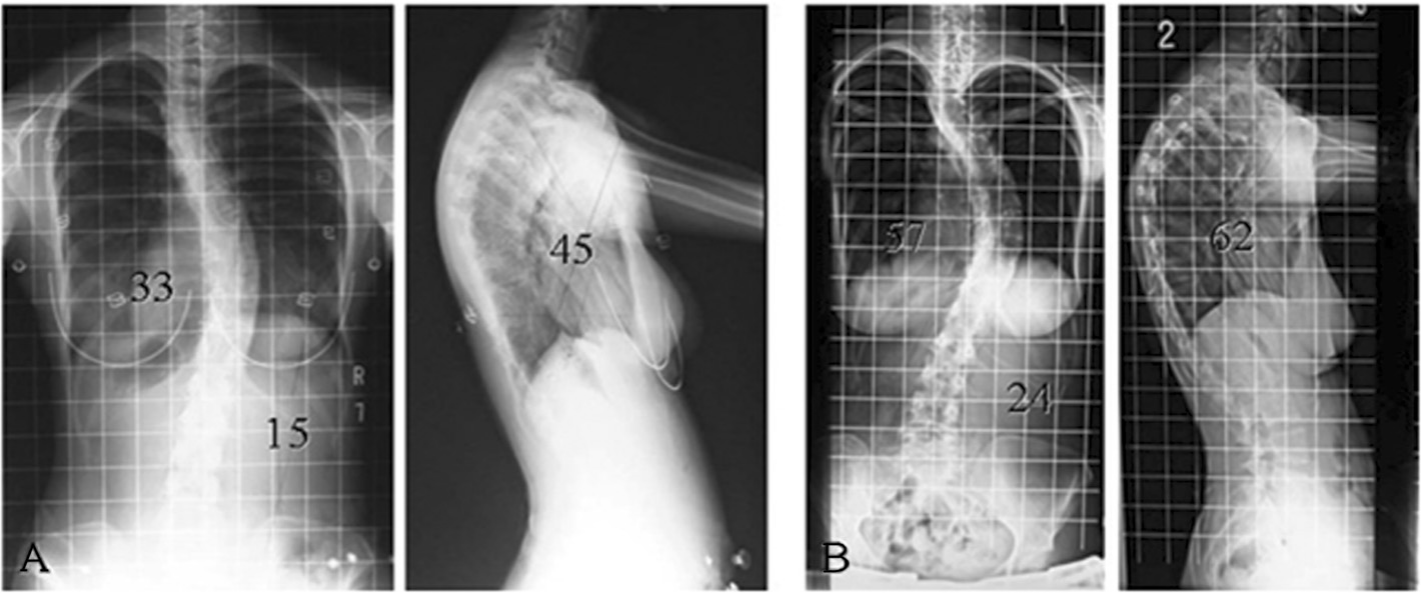
**Standing radiographs. (A)** Radiographs 1 month after implant removal. **(B)** Radiographs 18 months after implant removal.

Implant removal was performed, but part of the sublaminar wiring could not be removed due to solid bony union surrounding the wires. Intraoperative exploration of the fusion mass revealed no evidence of pseudarthrosis. One month after removal, her right thoracic curve was 33°, representing an additional 4° loss of correction (Figure 2A). The patient failed to follow up, and we had no further contact with her until she returned for care 18 months after implant removal after noting a recurrence of her deformity.

**Figure 3:**
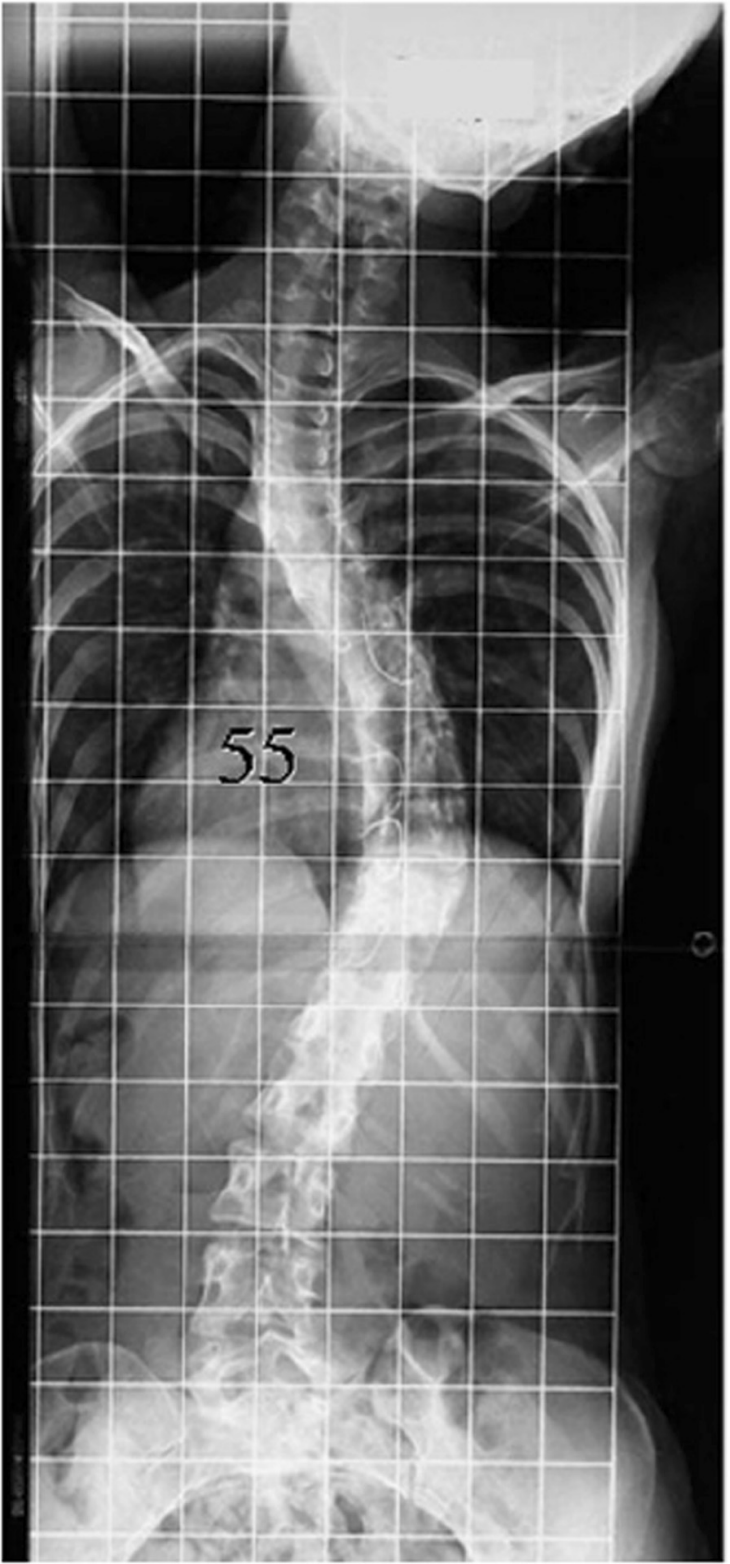
Bending radiograph prior to reinstrumentation.

**Figure 4:**
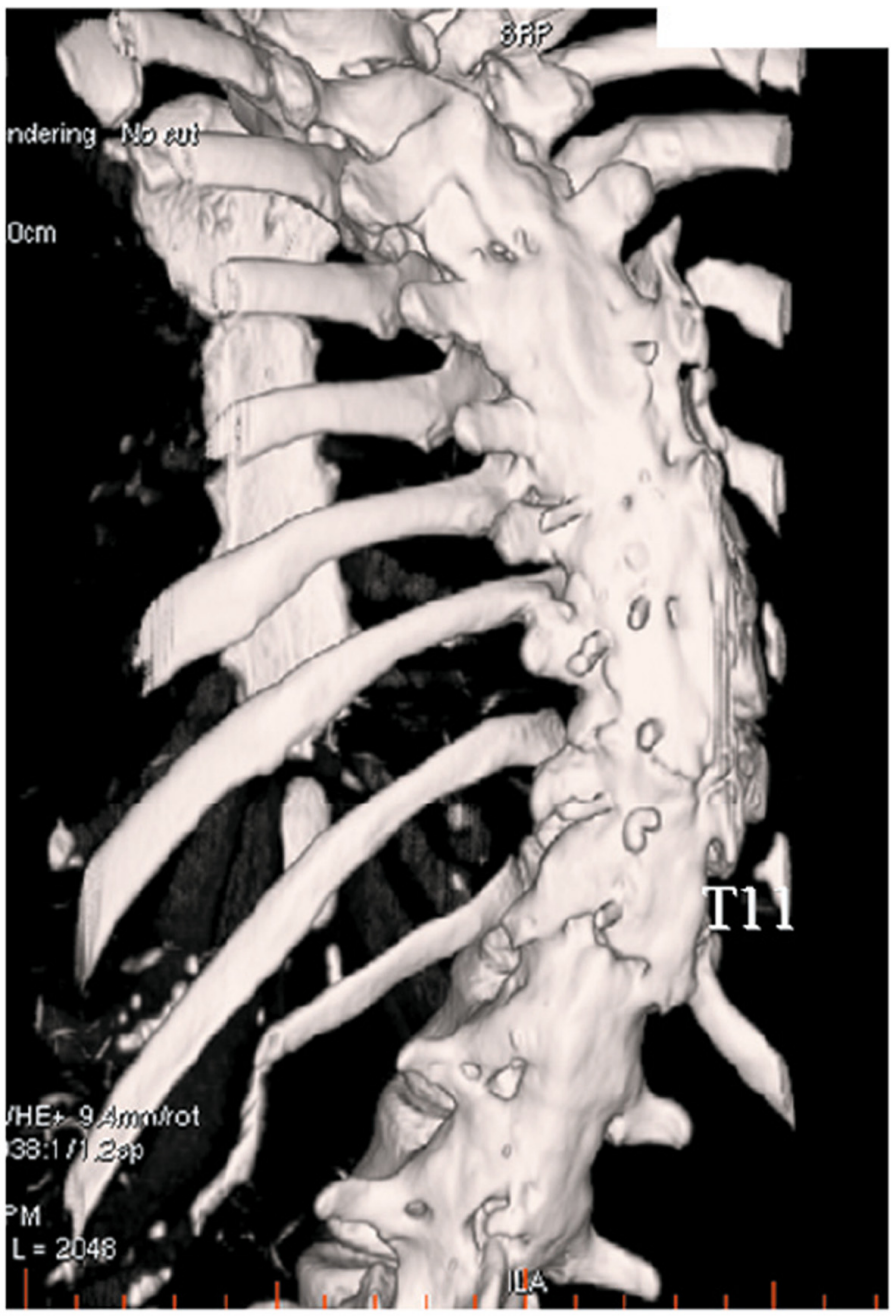
**Three-dimensional computed tomography (CT) image prior to reinstrumentation.**

Comparison of radiographs at that time with radiographs prior to implant removal demonstrated significant progression of her thoracic curve, from 29° to 57°. The kyphosis between T5 and T12 also had increased, from 45° to 62° (Figure 2B). She had no neurologic deficits since the time of the initial instrumentation. On examination, the thoracic curve demonstrated minimal flexibility on preoperative bending films (Figure 3). Preoperative three-dimensional CT scanning revealed partial clefts between T11 and T12, but the CT images were inconclusive as to whether the fusion mass had a complete lack in continuity or non-union (Figure 4). She was assumed to have solid fusions at this time.

### Surgery

**Figure 5:**
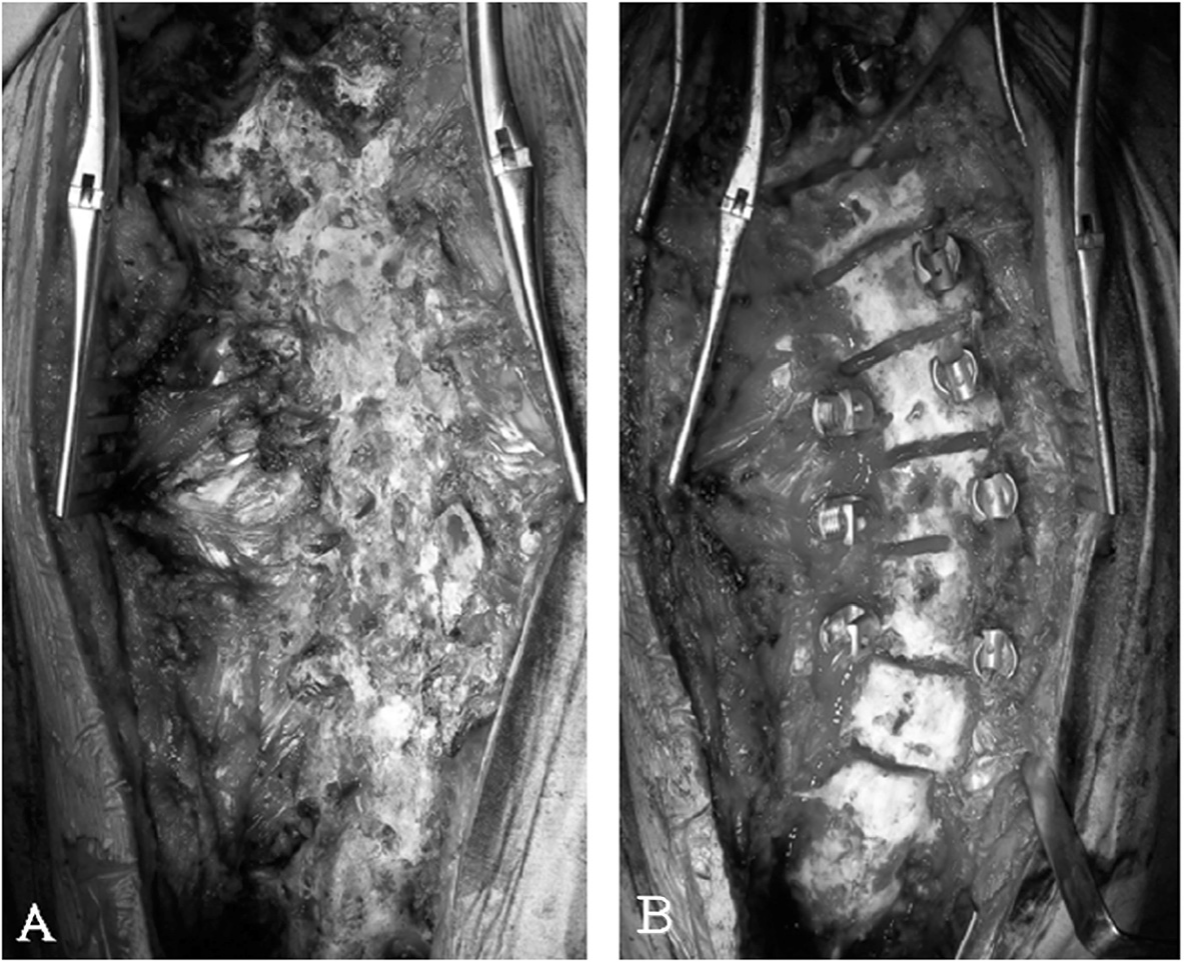
**Operative photographs.** Intraoperative view **(A)** before osteotomies and **(B)** after osteotomies.

**Figure 6:**
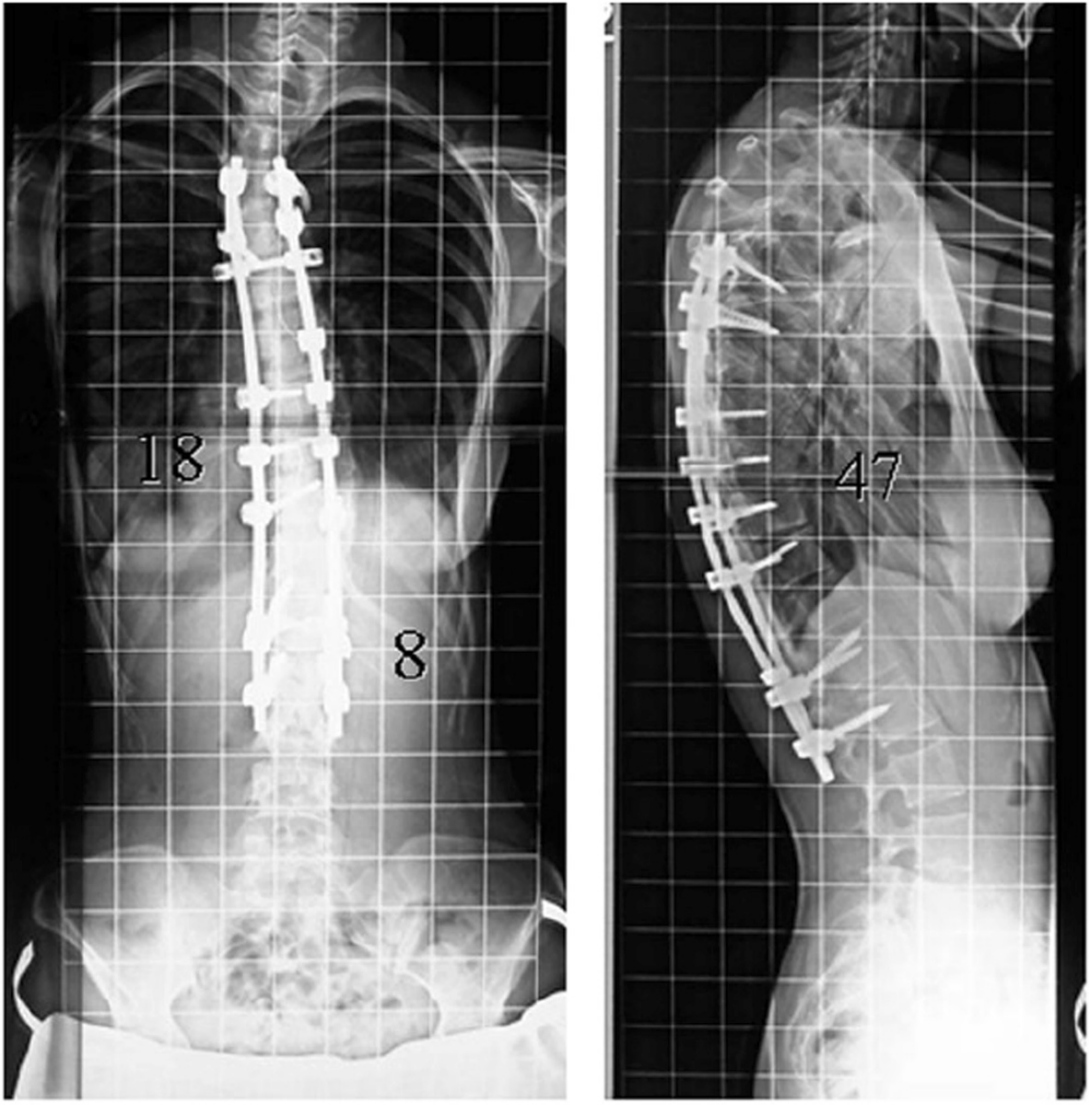
Radiographs after reinstrumentation surgery.

During the third operation, the fusion mass was completely exposed and explored (Figure 5). Although no pseudarthrosis had been identified initially, more meticulous exploration using a nibbler and rongeur discovered left partial clefts of the fusion mass at T11–T12 above her previous posterior fusion, though no motion within the fused segments was detected. No other site of possible pseudarthrosis was found. Because of its rigidity, the deformity required Smith-Peterson osteotomy of the fusion mass and revision posterior fusion with instrumentation using 6.3 mm titanium rods at T5-L2. A CT-based navigation system was used to perform the osteotomy and to insert pedicle screws due to the lack of normal anatomical structures. The surgery reduced her scoliosis to 18° (Figure 6).

## Discussion

The results of instrumentation removal following posterior spinal fusion for treatment of AIS remain controversial. Rathjen *et al*. [5] studied 43 patients who had undergone posterior spinal fusion for idiopathic scoliosis and subsequently had complete removal of all instrumentation. They reported two patients with a progression of their coronal curve exceeding 10° (11° and 20°). Potter *et al*. [6] reported an immediate loss of approximately 4° (range 0°-8°) after removal, with continued settling of an additional 6° (10° total) in the main thoracic curve in adolescent idiopathic scoliosis. Helenius *et al*. [7] reported on 78 patients with adolescent idiopathic scoliosis after Harrington Instrumentation. Fifty of these patients underwent routine rod removal two years after spinal fusion. The mean preoperative, two-year follow-up, and 20-year follow-up thoracic Cobb angles were 53°, 40°, and 48°, respectively, in the patients who had implants removed. Muschik *et al*. [8] reported that radiographic follow-up revealed significant loss of correction *versus* the measurements obtained immediately before instrumentation removal in all 45 patients. Mean correction loss was 6° (from 31° to 37°) for the primary (thoracic) curve. In contrast, 15 months after removal, the main thoracic curve in the present patient increased from 29° to 57°. Surprisingly, her coronal curve fifteen months after removal was just as large as her initial, preoperative one (56°). This amount of progression far exceeds the reported data in both magnitude and rapidity of curve progression.

Several possible explanations could account for the amount of coronal curve progression following implant removal. Preoperative CT prior to reinstrumentation revealed partial clefts between T11 and T12, but no clear evidence of a pseudarthrosis. The partial cleft in the second reinstrumentation surgery showed one potential pseudarthrosis that had not been recognized at the time of surgical exploration during the removal surgery. The patient additionally might have had other occult pseudarthroses, despite the thoracic curve having demonstrated minimal flexibility within the fused area. Initially, the pseudarthrosis might have been covered by a weak sheet of bone similar to a mature fusion bone such that no motion was identified within the fused segments after removal. If we had subsequently removed the weak sheet of bone more, the pseudarthrosis might have manifested. Deckey *et al*. [9] reported that surgical exploration is imperfect for positive detection of a pseudarthrosis. CT, flexion/extension radiographs, and/or bone scintigraphy may be helpful to confirm the location and morphology of established nonunions, but current radiographic techniques are also inadequate for reliably excluding the presence of pseudarthrosis [10]. In previous reports, most of the authors identified no patient who was found intraoperatively to have a pseudarthrosis. The cleft in our case is not incompatible with these reports: because there is no definitive method for detecting pseudarthrosis, some of the patients in these prior studies may have had occult pseudarthrosis that was not detected. To our knowledge, no prior published reports have described a rapid worsening of the coronal curve in AIS as severe as that observed in the present patient. One possible explanation is that our patient might have had undetected pseudarthrosis covered by a weak sheet of bone similar to a mature fusion bone. Even if our patient had undetected pseudarthrosis, the observed amount and rapidity of curve progression is still unusual. Lack of good quality and quantity of autologous bone at the first surgery might have affected the lack of spinal fusion. Alternatively, the amount of fusion in our patient may have played a role in the coronal curve progression. Compared to pedicle screw fixation, which has been widely used in recent years, upper hook instrumentation with autogenous rib and local bone grafting might not have been strong enough to produce solid bone union. The thin layer evidently had enough strength to prevent mobility, requiring an osteotomy to correct, but possibly its strength was insufficient to resist the continuous load placed upon the spine, leading to coronal progression. Moreover, partial facetectomy in the first surgery might have created conditions resulting in worse effects compared with the natural history of uninstrumented scoliosis. Potter *et al*. [6] suggest that some differences between patients in curve settling reflect the degree of fusion mass consolidation or maturation. Usually, the increase in curvature that occurs is well-tolerated clinically [6], but in our patient who developed the worst reported increase in curvature, the coronal and sagittal curves became symptomatic, and notable cosmetic deformity recurred. One possible mechanism of the correction loss is that rebalancing the shoulders by a postural control system might increase the left proximal thoracic curve and secondarily the right thoracic main curve. This is speculation, however, and further study is required to establish the pathogenesis.

## Conclusions

We have presented herein a patient who, during implant removal, was assumed to have solid fusions but nonetheless developed coronal and sagittal worsening that eventually required reinstrumentation. Though there is no definitive method for detecting pseudarthrosis before removal surgery, we need to evaluate the quality of spinal fusion using CT scans or bone scintigraphy. Intraoperatively, we should check spinal fusion and perform re-fusion with iliac graft or bone substitutes. We feel that postoperative brace immobilization may also be necessary. In such cases, we need to carefully explain to patients that removal of their implants entails a risk of progressive deformity and that they need to follow-up with their physician after implant removal.

## Consent

Written informed consent was obtained from the parents of the patient for publication of this case report and any accompanying images. A copy of the written consent is available for review from the Editor-in-Chief of this journal.

## Competing interests

The authors declare that they have no competing interests.

## Authors’ contributions

TK performed surgeries, conceived the study and drafted the manuscript; SM performed surgeries, and performed critical revision of the manuscript and gave final approval of the version to be published; TA and TS performed surgeries, and collected and reviewed clinical and radiographic charts. All authors read and approved the final manuscript.
